# Water immobilization by glass microspheres affects biological activity

**DOI:** 10.1038/s41598-018-28123-4

**Published:** 2018-06-27

**Authors:** A. G. Marangoni, M. S. Al-Abdul-Wahid, R. Nicholson, A. Roma, A. J. Gravelle, J. De Souza, S. Barbut, P. A. Spagnuolo

**Affiliations:** 1 Department Food Science, University of Guelph, Guelph, ON N1G2W1 Canada; 2 NMR Centre, University of Guelph, Guelph, ON N1G2W1 Canada

## Abstract

We recently reported that the water holding capacity of myofibrillar protein hydrogels could be increased upon addition of small amounts of microparticles, particularly glass microspheres. Glass microspheres were found to decrease the spin-spin relaxation time (*T*_2_) of water protons in the gels, which was interpreted as enhanced water binding by the glass. We were thus interested in determining whether the observed effects on water proton relaxation were a direct consequence of water-glass interactions. Here we show how glass microspheres reduce the mobility of pure water, reflected in large decreases in the *T*_2_ of water protons, decreases in the self-diffusion coefficient of water molecules, a lower water activity, and strengthening of *O-H* bonds. Even though glass is considered an inert material, glass microspheres were shown to inhibit the growth of human embryonic kidney cells, and stimulate or inhibit the growth of leukemia and monocytic lymphoma cells *in vitro*, depending on dose and time. The germination of alfalfa seeds and the growth of *E.coli* cells were also inhibited upon exposure to glass microspheres. This work indicates that the properties and behavior of materials, even ones considered inert, can be affected by their size. These observations suggest possible toxicological consequences of exposure to microparticles, but also open us possibilities to affect cellular/organism function via modulation of macromolecular hydration.

## Introduction

**Figure 1:**
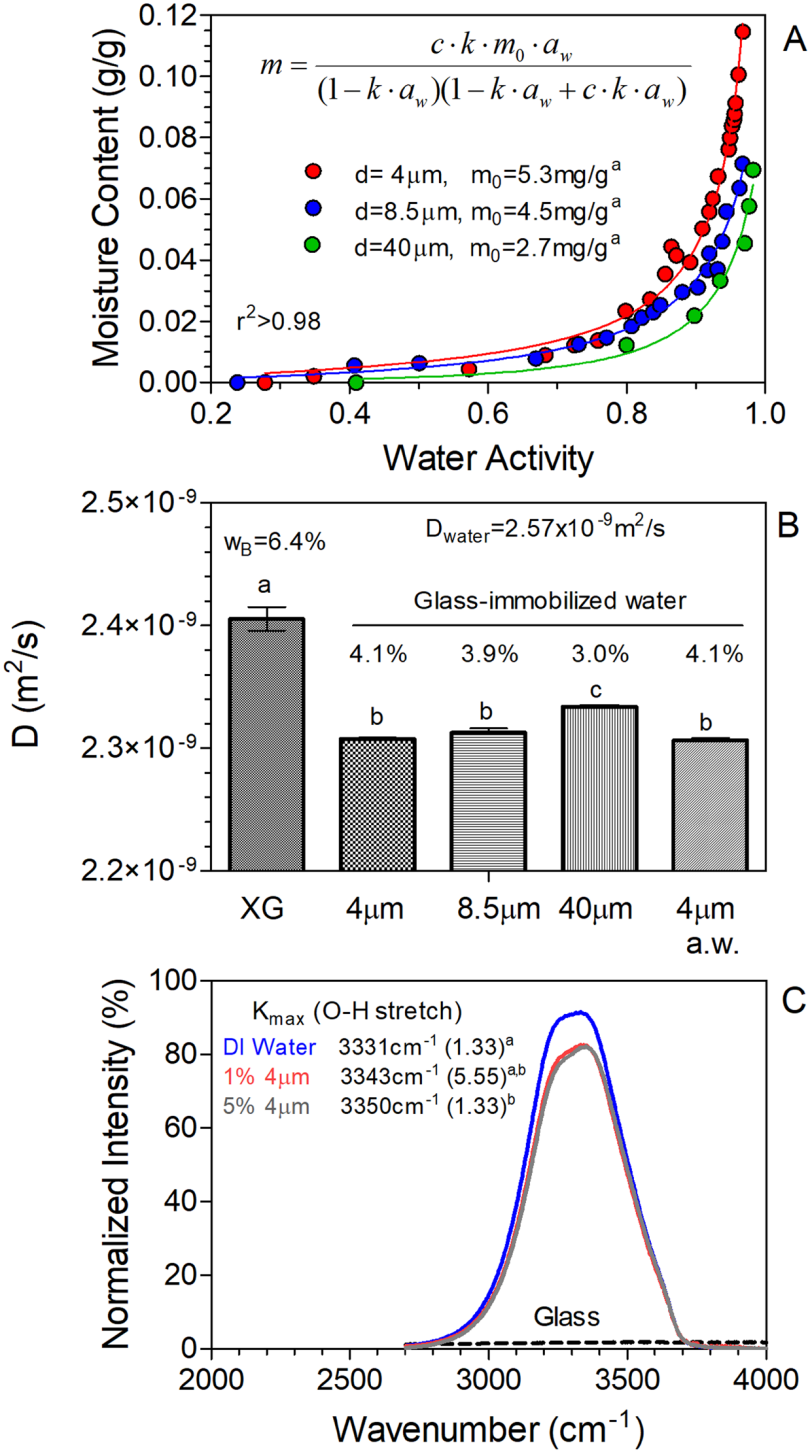
(**A**) Water activity determined for glass bead dispersions with increasing water content for glass microspheres of varying size; 4 μm (red), 8.5 μm (blue), and 40 μm (green). Bound moisture (m_0_) was calculated using the GAB isotherm model, using the equation shown. Each data point represents a different sample. (**B**) Water diffusion coefficient measured for a 0.5% xanthan gum solution (XG) in deionized water, and XG solutions with dispersed glass microspheres (1% v/v) and acid-washed (a.w.) 4 μm microspheres. The diffusion coefficient of deionized water (no XG) is shown at top of panel. Percentages above bars indicate corresponding amounts of bound water based on relative decreases in the diffusion coefficient. (**C**) FTIR O-H stretching band of pure deionized water (blue) and glass bead dispersions (1% v/v – red, 5% v/v – gray). Peak positions are denoted in legend, and signal from glass powder is shown with black dashed line.

We recently reported that the water holding capacity of myofibrillar protein hydrogels could be increased upon addition of small amounts of microparticles^1^, particularly glass microspheres^2,3^. Glass microspheres were found to decrease the spin-spin relaxation time (*T*_2_) of water protons in the myofibrillar gels, which can be interpreted as enhanced water binding by proteins or glass^4^. We were thus interested in determining whether the observed effects on water proton relaxation were a direct consequence of water-glass interactions. A common starting point in water-binding studies is the generation of a binding isotherm through measurements of water activity at different water contents^5^. Figure 1A shows such isotherms for 4, 8.5 and 40 μm diameter glass microspheres. The trends obtained were as expected, with the smaller diameter, *i.e*., greater surface area, particles displaying the highest monolayer coverage value, and thus lowest water activity (Fig. 1A). Should significant differences in bound water arise due to the presence of glass microspheres, differences should also be detected in the self diffusion coefficient (*D*) of water. Diffusion NMR experiments showed that the *D* of water decreased significantly (P < 0.05) upon addition of glass microspheres to a xanthan gum solution (Fig. 1B). Xanthan gum was added to prevent sedimentation of the glass microparticles, and itself decreased the reported diffusion coefficient of water^6^ by 6.4% (P < 0.05). The *D* was similar for 4 and 8.5μm diameter glass microspheres (P > 0.05) but higher for the 40μm microspheres (P < 0.05).

The population of total bound water can be estimated from the NMR-derived diffusion coefficients. Assuming that the exchange between free and bound water is fast on the timescale of the diffusion experiments (50 msec), and that the addition of the glass particles does not affect the local viscosity of free water molecules, the observed diffusion coefficients are a population-weighted average of the free and bound water diffusion coefficients (*i.e*., *D* = *p*_*free*_*∙D*_*free*_ + *p*_*bound*_*∙D*_*bound*_). The population of total bound water may be calculated assuming *D*_*free*_ is equal to that of water in a xanthan gum solution, while *D*_*bound*_ values are estimated via the Stokes-Einstein relation assuming 4, 8.5 and 40 μm particle diameters. Estimates of the amount of total bound water are reported in Fig. 1B, which was found to increase with decreases in glass microsphere diameter (P < 0.05). We then proceeded to estimate the amount of multilayer bound water in the system from the difference between total bound water determined by NMR diffusion experiments and monolayer surface bound water determined from water activity measurements (Figure S1). Estimates of monolayer surface bound water are relatively straightforward to obtain from fits to moisture sorption isotherm models; however, the more ill-defined multilayer bound water, described as layers of progressively less immobilized water from bound surface monolayer water to bulk free water, is difficult to determine. Here we show a promising approach to determining the amount of this multilayer water, which may help quantify this dynamic component of water binding.

The infrared spectrum of water was also significantly affected by addition of 4μm glass microspheres (Fig. 1C). A statistically significant 19 cm^−1^ increase (P < 0.05) in the peak wavenumber (*K*_*max*_) for the *O-H* stretching vibration was interpreted as a strengthening of the *O-H* bonds of water based on the relationship between peak wavenumber (*K*) and the force constant of a bond (*f*), namely $f\approx {(2\pi cK)}^{2}$, where *c* is the speed of light. On the other hand, the melting point, melting entropy and enthalpy of water, as determined by differential scanning calorimetry, remained unchanged in the presence of 5% (v/v) 4 μm microspheres (not shown).

**Figure 2:**
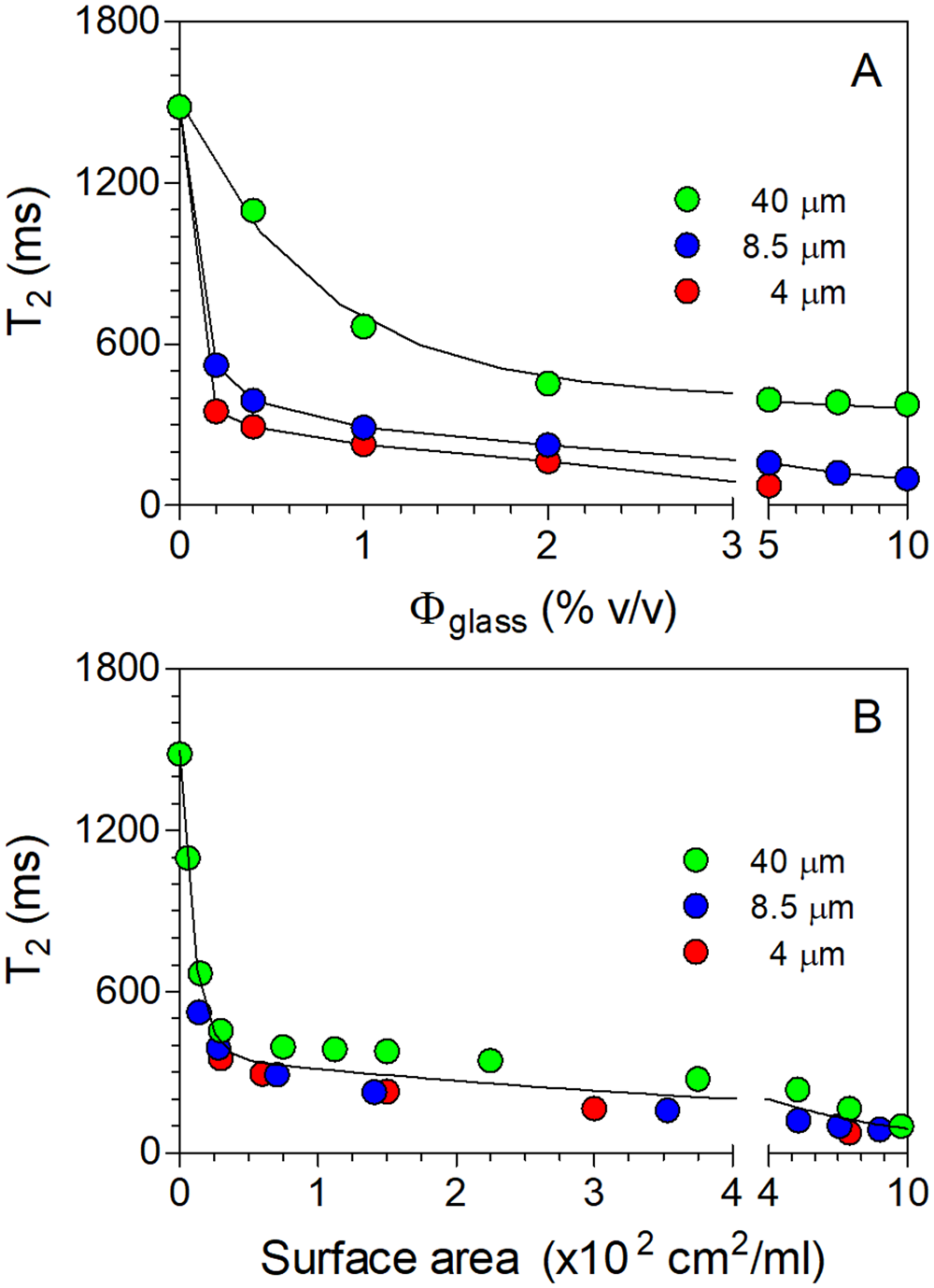
Spin-spin relaxation time (T_2_) for suspensions of glass microspheres of different sizes as a function of volume fraction present (**A**), and corresponding surface area (**B**). All suspensions contained 0.5 wt% xanthan gum in deionized water. Experiments were performed at a 20 MHz NMR field strength.

Since glass microspheres induced decreases in the *T*_2_ of water in myofibrillar hydrogels^2,3^, we decided to try it on systems containing only glass microspheres in deionized water. Figure 2A shows the decrease in the *T*_2_ (measured at 20 MHz NMR field strength) of water protons in a 0.5% xanthan gum solution upon addition of glass microspheres of three different diameters. A large drop in *T*_2_ from ~1500 ms to ~250 ms was observed upon addition of only 1% (v/v) 4μm glass microspheres. The smaller the particles, the greater were the observed effects, in a concentration-dependent manner (Fig. 2A). The large decrease in the observed *T*_2_ is due to the exchange of the water molecules between two (or more) chemical environments. In systems with chemical exchange, the observed relaxation rate, *R*_2_ (=1/*T*_2_), is a sum of the *R*_2_ in the absence of exchange, and an exchange contribution. The exchange component can dominate the observed *R*_2_ values in this situation. Decreases in *T*_2_ measured at 300 MHz field strength were even larger (Table S1).

By plotting all *T*_2_ data as a function of the amount of glass surface area in the system (Fig. 2B), it became obvious that the effect was solely dependent on the available amount of surface area for interaction with water molecules. We interpreted this as water molecules interacting with the glass surface, consistent with infrared spectroscopy results (Fig. 1C). The spin-lattice relaxation time (*T*_1_) of water followed similar trends (Figure S2), but the magnitude of the decrease was smaller. As expected, decreases in *T*_2_ were correlated to decreases in *D* (Figure S3A), where a decrease in D is associated with an increase in the amount of bound water. This increase in the amount of bound water was correlated with an increase in the monolayer amount of bound water using water activity measurements (Figure S3B). *T*_2_ measurements are ideal for the study of surface interactions of water and can provide insight on chemical exchange^7^. Results obtained in this study are consistent with atomic scale molecular dynamics computer simulations and experimental results of water molecules on amorphous silica surfaces^8,9^. These studies revealed the arrangement and interactions of water molecules at the silica interface and their effect of NMR relaxation, water diffusivity and IR spectra.

To further characterize the observed water interaction with glass surfaces, we carried out relaxation dispersion measurements by pulsed NMR. The *R*_2_ values obtained at different inter-pulse spacings (τ_CP_) will be constant if there is no chemical exchange, and will vary with τ_CP_ if there is chemical exchange between bound and free water. A plot of the *R*_2_ of water protons for our three systems as a function of 1/τ_CP_ indicated exchange of water protons between the surface of the glass and the bulk (Figure S4).

*T*_2_ values decreased linearly with increasing temperature for suspensions of glass in 0.5% xanthan gum (Figures S2 and S5), while the opposite was observed for the *T*_2_ of pure water and *T*_1_ values (Figures S2 and S6). Again, this effect was solely dependent on the amount of glass surface area present in the suspensions (Figure S7). Such *T*_2_ thermal behavior has been previously reported for pure water and silica-water systems where observed *T*_2_ values are dominated by exchange contributions ^7,10^. The *T*_1_ behavior is also consistent with previous reports^7,10^.

To eliminate the possibility of a paramagnetic impurity being present in our sample and affecting results, we centrifuged the samples containing the glass microspheres and measured *T*_2_ of the supernatants. The values were not significantly different from those of deionized water (Table S2). We were also concerned about pH-related effects and thus proceeded to acid wash the glass microspheres to determine if the soda-lime nature of the glass influenced pH and indirectly water mobility at the glass surface. Results shown in Table S1 show that acid washing did not affect the measured *T*_2_ of water. We also measured the surface charge of the glass microspheres, which was ~−40 mV in the pH range 2–8, and did not change with acid washing (Figure S8). Maybe the presence of this surface charge explains the apparent lack of aggregation of these glass microspheres at the low concentrations used, as determined by static light scattering (Figure S9).

Since water is such an integral part of living biological matter, subtle changes in its dynamics could possibly have significant effects on structure and function of biological systems. We thus proceeded to explore the effects of glass microspheres on the growth of human embryonic kidney cells, leukemia and monocytic lymphoma cells, *E.coli*, and on the sprouting of alfalfa seeds.

**Figure 3:**
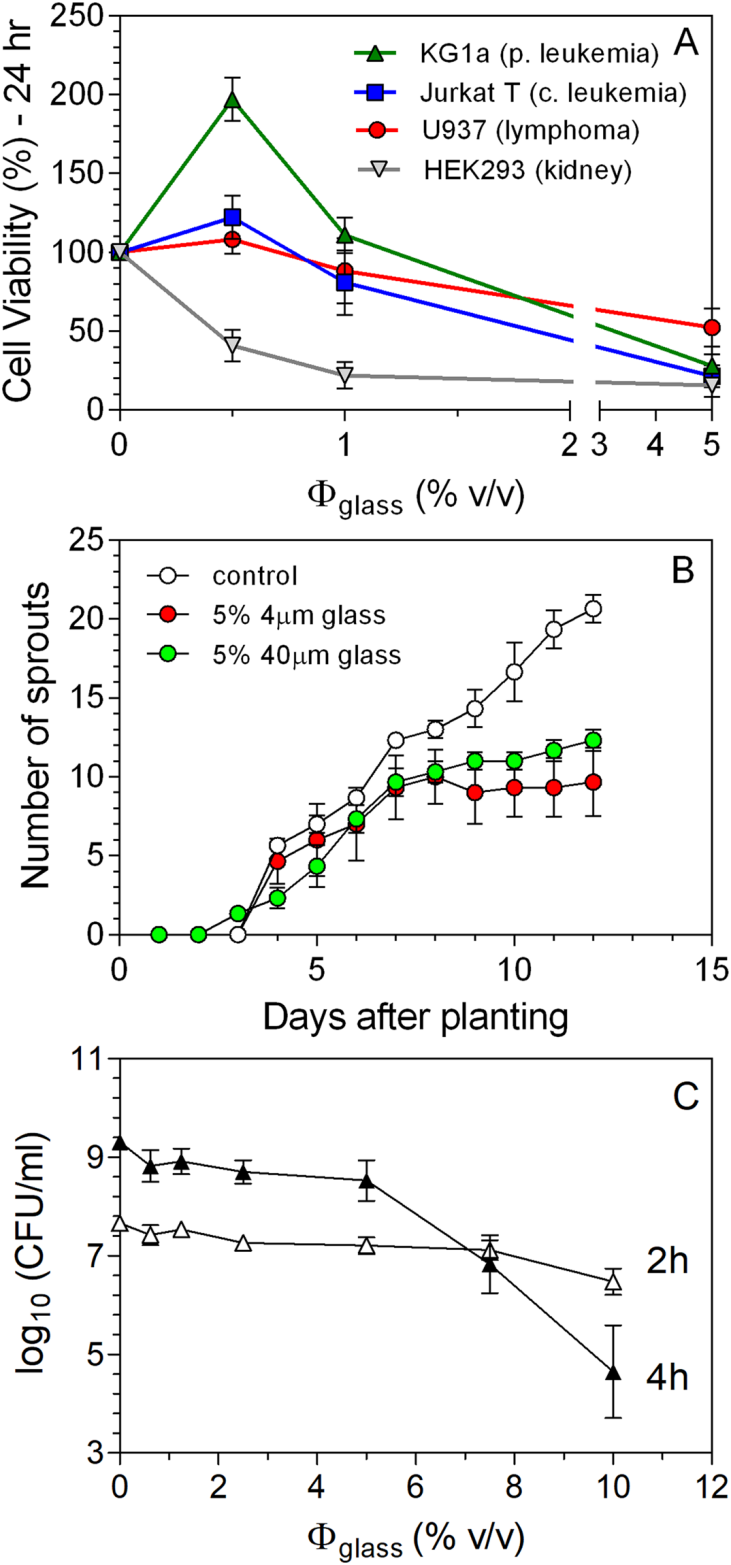
(**A**) Viability of human lymphoma, leukemia and embryonic kidney cell lines after a 24 hr incubation period in media supplemented with 4 μm glass microspheres (denoted as % v/v glass). (**B**) Number of sprouts emerging from alfalfa seed grown in deionized water (white), and water supplemented with 5% (v/v) 4 μm (green) or 40 μm (red) acid washed glass microspheres. Each sprouting dish contained 40 seeds which were grown over a 12-day period. (**C**) Impact of supplementing growth medium with 5% (v/v) 4 μm glass microspheres on the viability of E. coli BL21 cells (reported as colony forming units per ml; CFU/ml), after 2 hr or 4 hr incubation periods.

The growth of embryonic kidney cells after 24 hr was severely inhibited by the presence of even only 0.5% (v/v) 4 µm glass microspheres in the growth medium (Fig. 3A). The growth of both the leukemia and lymphoma cells was also inhibited, although less drastically, above 1% (v/v) levels. Surprisingly, though, cell viability was enhanced for the acute promyelocytic leukemia cell line (KG1a) after 24 hr exposure to growth medium containing 0.5% (v/v) glass microspheres (Fig. 3A), but not for cell leukemia (Jurkat T) or monocytic lymphoma (U937) cell lines. This would indicate a possible activation of tumor cell growth for certain cell lines at specific levels of 4 µm glass microsphere exposure. The stimulation effects were, however, transient, and the activation of KG1 was not observed after 48 hr of exposure to the glass microspheres (Figure S10B). The kidney cells remained fully inhibited regardless of incubation time. The mechanism responsible for the different sensitivity of the cells to glass microsphere addition remains unknown.

The germination of alfalfa seeds was strongly affected by the presence of 4 µm and 50 µm glass microspheres in the water, both in terms of the number of sprouts (Fig. 3B and the number of leaf pairs (Figures S11) (P < 0.001). This effect was concentration dependent, with saturation reached at 0.5% (v/v) addition levels (Figure S12). Acid washing of the glass microspheres did not affect any of these results either. We were also concerned that glass was binding minerals, making them less accessible for sprout growth. We therefore sprouted seeds in a standard plant tissue culture growth medium as well. The effects were like those in deionized water (Figure S13). The growth of the prokaryotic organism *E. coli* was also significantly (P < 0.05) affected after 4 hr exposure during growth in tryptic soy medium in the presence of 4um glass microspheres (Fig. 3C). It is possible that water binding could be affecting the hydration of structural proteins, enzymes, membranes and other supramolecular structures in biological systems, which affects their function^4,11^.

Glass microspheres are widely used in the powder composites industry and as additives in paint and cosmetics for shine. Glass is usually considered an inert substance. However, our results suggest that a reduction in the size of glass in the low micrometer range might have an effect on biological structures and their function, possibly mediated through interactions with water. It is plausible that microparticles derived from many materials (Table S3) may have similar effects on biological systems. Moreover, the ability to affect the dynamics of water using glass microspheres opens possibilities for affecting the hydration and function of macromolecular structures in living systems.

## Experimental Methodology

### Glass Microspheres

Three sizes of glass microspheres were used throughout the experiments: 4 µm (Solid Soda Lime Microspheres, Cospheric LLC, Santa Barbara, CA), 7–10 µm, and 30–50 µm (Spheriglass Solid Glass Microspheres, Potters Industries LLC, Valley Forge, PA).

### Glass Microsphere Size Distribution Analysis

The particle size distributions of the glass microspheres were measured using a light scattering device (Mastersizer 2000, Malvern Instruments Ltd., UK) equipped with a small volume sample dispersion unit (Hydro 2000SM). Samples of each of glass size were first mixed in a separate beaker of DI water to ensure minimal clumping. This mixture was then added to the DI water in the dispersion unit. Stirring was set at 1600 rpm and a refractive index of 1.52 was used to determine each particle size distribution. Analyses were carried out in triplicate.

### NMR Spectroscopy

NMR relaxation experiments were collected on a Bruker AVANCE spectrometer with a ^1^H operating frequency of 300 MHz, using a 5 mm BBFO probe. NMR diffusion experiments were collected on a Bruker AVANCE spectrometer with a ^1^H operating frequency of 600 MHz, using a 5 mm TXO probe. The sample temperature was regulated at 25.5 ± 1 °C unless otherwise specified. In all cases, spectra were baseline corrected with a polynomial function prior to integration of the water peak (I_water_).

T_1_ measurements were acquired using a saturation recovery sequence. Generally, the water resonance was saturated for 10 seconds using a 50 Hz continuous wave decoupling field followed by a relaxation delay period (τ) and a pulse to measure the signal intensity after relaxation. Ten spectra, with relaxation delays (RD) ranging from 0.1 to 8.0 sec, were collected in a random order. T_1_ values were calculated via a non-linear-least-squares fit of:

1 $${{\rm{I}}}_{{\rm{water}}}={{\rm{I}}}_{{\rm{f}}}(1-\exp ((-{\rm{RD}}+{\rm{\alpha }}){/{\rm{T}}}_{{\rm{1}}}))$$

where I_f_ and α are fitted parameters representing the water peak area at infinite relaxation delay and a correction factor for imperfect saturation pulses, respectively.

T_2_ measurements were acquired using a CPMG pulse sequence. The spacing between consecutive 180° pulses was fixed at 2 msec for all experiments in order to mitigate any difference arising from exchange effects. Nine spectra, with relaxation delays ranging from 0.032 sec to 0.512 sec were collected in a random order. T_2_ values were calculated via a non-linear-least-squares fit of:

2 $${{\rm{I}}}_{{\rm{water}}}={{\rm{I}}}_{{\rm{0}}}(\exp (-{\mathrm{RD}/{\rm{T}}}_{{\rm{2}}}))$$

where I_0_ is a fitted parameter representing the water peak area at zero relaxation delay.

T_2_ relaxation dispersion measurements (aka CPMG-RD) were acquired using a CPMG pulse sequence in which both the number of 180° pulses and the spacing between them (τ_cp_) were jointly varied such that the total relaxation time (BigT) was always 0.192 sec (for 4 micron and 7–10 micron samples) or 0.384 sec (for 30–50 micron samples). Twenty-seven spectra, with τ_cp_ values ranging from 1 msec to 96 msec, were collected in a random order; also collected was one spectrum with zero relaxation delay (I_noCP_). For each τ_cp_ value, the corresponding T_2_(τ_cp_) was calculated by:

3 $${{\rm{T}}}_{{\rm{2}}}({{\rm{\tau }}}_{{\rm{cp}}})=-\,1/{\mathrm{BigT}}^{\ast }{\mathrm{In}(I}_{{\rm{water}}}({{\rm{\tau }}}_{{\rm{cp}}}){/I}_{{\rm{noCP}}})$$

Plots of the measured T_2_(τ_cp_) against 1/τ_cp_ provide a qualitative measure of the degree of exchange: flat plots indicate suggest no exchange (or, similarly, infinitely fast exchange), while plots which decay with increasing 1/τ_cp_ suggest exchange between free water and a putative ‘glass-associated’ species.

Diffusion measurements were performed using a bipolar pulse pair stimulated echo (BPPSTE) sequence [Wu, D.H., Chen, A.D., Johnson, C.S. 1995. An Improved Diffusion-Ordered Spectroscopy Experiment Incorporating Bipolar-Gradient Pulses. Journal of Magnetic Resonance, Series A 115 (2): 260–264] as provided in the Bruker pulse sequence library (sequence file *stebpgp1s*).

Signal intensity in the BPPSTE experiment is given by Wu *et al*. (1995):

4 $$S({\rm{g}})={\rm{S}}({\rm{0}})\exp [\,-\,{D({\rm{\gamma }}g{\rm{\delta }})}^{{\rm{2}}}({\rm{\Delta }}-{\rm{\delta }}/3-{\rm{\tau }}/2)$$

where s(0) is the intensity in the absence of encoding/decoding gradients, D is the diffusion coefficient, γ is the gyromagnetic ratio, g is the gradient strength, δ/2 is the duration of each gradient pulse, Δ is the overall diffusion time, and τ is a small correction factor associated with the BPPSTE sequence.

The integral of the water resonance (S(g)) was measured for 35 different gradient strengths ranging from 2.6 to 32.3 G/cm. The diffusion coefficient of water was calculated from a non-linear-least-squares fit of these intensities to eq. [4], with experimental parameters held fixed at their respective values of δ = 2 msec, Δ = 50 msec, and τ = 0.22 msec. S(0) was not fixed during the fitting process.

### Water Activity

Glass microspheres were dried at 120 °C for 48 hours and cooled in a desiccator prior to testing to ensure that any residual moisture was removed from the surface of the glass. Approximately 3 g of each particle size range were transferred into a disposable sample trays and water activity was measured using an Aqua Lab Dew Point Water Activity Meter 4TEV (Decagon Devices Inc., Pullman, WA). A known amount of DI water was added drop wise to the glass microspheres and the samples were covered and left to equilibrate for 30 min before each measurement at 25 °C. Water activity vs. water content data was fitted to the GAB model using GraphPad Prism 5.0 software (La Jolla, CA, USA) to derive estimates of monolayer bound water.

### Differential Scanning Calorimetry (DSC)

DSC was performed on three replicates of DI water, as well as a 0.05 volume fraction dispersion of the 4 µm glass microspheres in DI water using a DSC 1 instrument (Mettler-Toledo, Mississauga, ON, Canada). The glass bead and water mixtures were mixed immediately prior to testing. 4–6 mg of sample was placed in an aluminum DSC pan, capped and hermetically sealed. Samples were cooled from 25 °C to −25 °C and subsequently heated to 40 °C, at a rate of 5 °C/min. Peaks were analysed using Star Software (Mettler-Toledo) provided with the DSC instrument.

### Fourier Transform Infrared (FTIR) Spectroscopy

FTIR spectra were recorded using a FTIR spectrometer (Nicolet Nexus 470, Thermo Electron, Waltham, MA, USA) equipped with a horizontal attenuated total reflectance (HATR) plate (Thermo Electron, Waltham, MA, USA). Three replicates of 0.00, 0.01, and 0.05 volume fractions of the 4 µm glass microspheres in DI water were performed. An average of 40 scans were collected for each sample. A background scan was performed between samples.

### Effects on mammalian cell growth

Jurkat T cell leukemia and U937 monocytic lymphoma cell lines were cultured in RPMI-1640 Medium (Thermo Fisher; Waltham, MA, USA) while the acute promyelocytic leukemia cell line, KG1a was cultured in Iscove’s Modified Dulbecco’s Medium (IMDM; Life Technologies; Grand Island, NY). Human embryonic kidney cells (HEK 293) were grown in DMEM Medium (Life Technologies). Media was supplemented with 10% fetal bovine serum (FBS) and antibiotic solution (Sigma Chemical) to obtain 200 µg of streptomycin and 200 units of penicillin per millilitre of media. All cells were maintained in an incubator at 37 °C with 5% CO_2_ and 95% humidity. For Jurkat, U937 and KG1a cells, 1 × 10^5^ cells/mL were seeded in a 10 mL culture dish in the presence or absence of beads at the indicated percentage (w/w). Similarly, HEK 293 cells were seeded at a concentration of 0.5 × 10^5^ cells/mL, incubated for 24 hours to allow for cell attachment and then beads were added. Following a 24-hour and 48-hour incubation, cells were then incubated with 20 µL of 3-(4, 5-dimethylthiazol-2-yl)-5-(3-carboxymethoxyphenyl)-2-(4-sulfophenyl)-2H-tetrazolium salt (MTS; Promega; Madison, WI) for two hours at 37 °C and 5% CO_2_. Metabolically active cells express extracellular enzymes which can cleave the MTS into a coloured formazan product. The formazan product was then quantified by measuring absorbance at 490 nm using a Biotek Synergy HT spectrophotometer (Biotek; Winooski, VT). Result are statistically analyzed using the GraphPad 5.0 prism software and expressed as a percent viability of the untreated control.

### Effects on Microbial Growth

A 20 mL sample of Tryptic Soy Broth (TSB) was mixed with increasing volume fractions (0.00, 0.00625, 0.0125, 0.025, 0.05, and 0.10) of 4 µm glass microspheres. These mixtures were inoculated with 1 mL of a culture of *E.coli* BL21. 0.1 mL of the culture was plated at time zero to establish a baseline. Inoculated TSB and glass bead mixtures were incubated at 37 °C for 2 h and 4 h, while being shaken at 120 rpm to keep the glass microspheres suspended. At both times, dilutions of each sample were made and 0.1 mL of each dilution was plated twice on Tryptic Soy Agar (TSA) plates. Plates were incubated for 24 h at 37 °C before being counted.

### Effects on Alfalfa Sprout Growth

12 g of DI water was mixed with the 4 µm glass microspheres at volume fractions of 0.00, 0.0025, 0.005, 0.01, and 0.05, as well as the 30–50 µm particles at a volume fraction of 0.05. Water and glass mixtures were transferred to plastic petri dishes, to which 40 alfalfa sprouting seeds (Country Creek Acres, Brentwood, MO, USA) were added. The dishes were topped up twice per day over eleven days to ensure that a consistent volume fraction of glass microspheres was maintained. Germination, tail growth, sprout growth, and leaf growth were recorded over the course of the experiment. Three replicates were performed for each volume fraction. For germination experiments in the presence of minerals instead of deionized water, Gamborg’s B-5 Basal Broth with Mineral Organics (Sigma Aldrich, St. Louis, MO, USA) was used (Gamborg, O.L., Miller, R.A. and Ojima, K. 1968. Nutrient requirements of suspension cultures of soybean root cells. Exp Cell Res. 50(1): 151–158).

### Replication

All experiments report the means and standard errors of three separate sample replicates, except for the mammalian cell growth studies, where nine separate separate samples were analyzed.

### Data availability

All raw data is available upon request in excel files. Please contact A.G. Marangoni (amarango@uoguelph.ca).

## Electronic supplementary material

Supplementary Information
