# Supplementary material for: Water immobilization by glass microspheres affects biological activity
Source: Sci Rep. 2018 Jun 27;8:9744. doi: 10.1038/s41598-018-28123-4 (PMC6021452; doi:10.1038/s41598-018-28123-4)
Supplement: Supplementary file 1 — Supplementary Information [file 41598_2018_28123_MOESM1_ESM.pdf]

Water immobilization by glass microspheres affects biological activity

A.G. Marangoni[a], M.S. Al-Abdul-Wahid[b], R. Nicholson[a], A. Roma[a], A.J. Gravelle[a], J. De Souza[a], S. Barbut[a], P. A. Spagnuolo[a]

[a]Dept. Food Science, University of Guelph, Guelph, ON, Canada N1G2W1

[b]Dept. of Chemistry, University of Guelph, Guelph, ON, Canada N1G2W1

Table S1 Comparison of water T_2 values for suspensions of glass microspheres (GB) suspended in 0.5 wt% xanthan gum (XG). For reference, the T_2 of deionized water determined in this study was 2680 ms. All measurements were conducted at 300 MHz NMR field strength, and a sample temperature of 298K. *Values are reported in milliseconds.

	0.5% XG Control	1% v/v 4µm GB	1% v/v 4µm GB Acid Washed	1% v/v 7µm GB	1% v/v 40µm GB
Rep 1	1110	91.4	92.3	170	748
Rep 2	1110	81	86	171	727
Rep 3	1116	84	84	161	725
Avg T_2	1112.0	85.5	87.4	167.3	733.3
STD DEV	3.46	5.35	4.33	5.50	12.74

Table S2 Comparison of water T_1 and T_2 relaxation values for pure deionized water, suspensions of 4µm glass microspheres in deionized water, and the supernatant from of a centrifuged dispersion of glass microspheres in water. All measurements were conducted at 300 MHz NMR field strength, and a sample temperature of 298K. *Values are reported in seconds.

	T1	T1.err		T2	T2.err
**Pure Water**	**3.46**	**0.01**		**2.68**	**0.02**
1% 4µm GB Rep 1	3.126	0.01281		0.1704	0.0048511
1% 4µm GB Rep 2	3.1217	0.0096687		0.1649	0.00573
1% 4µm GB Rep 3	3.1503	0.0081733		0.1647	0.004793
Average	3.133			0.167	
STD	0.015			0.003	
**Pure Water**	**3.46**	**0.01**		**2.68**	**0.02**
Supernatant 1	3.3846	0.0058125		2.6166	0.030996
Supernatant 2	3.3717	0.0059456		2.5366	0.027167
Supernatant 3	3.3715	0.0028084		2.6253	0.016862
Average	3.376			2.593	
STD	0.008			0.049	

Table S3 Comparison of water T_2 values for suspensions of various particles dispersed in 0.5 wt% xanthan gum at 0, 1% and 5% (v/v) concentrations. All measurements were conducted at 20 MHz NMR field strength. *Values are reported in milliseconds.

Volume fraction (v/v %)	0	1%	5%
Glass beads, 4µm	1490	245	66
Walnut shells	1490	800	220
Polyethylene microspheres, 4µm	1490	1407	1285
Diamonds, 4µm	1450	825	375*
Glass beads, 4µm	1450	240	72*

Note: Last two rows were collected previously, and * indicates actual volume fraction of 0.04

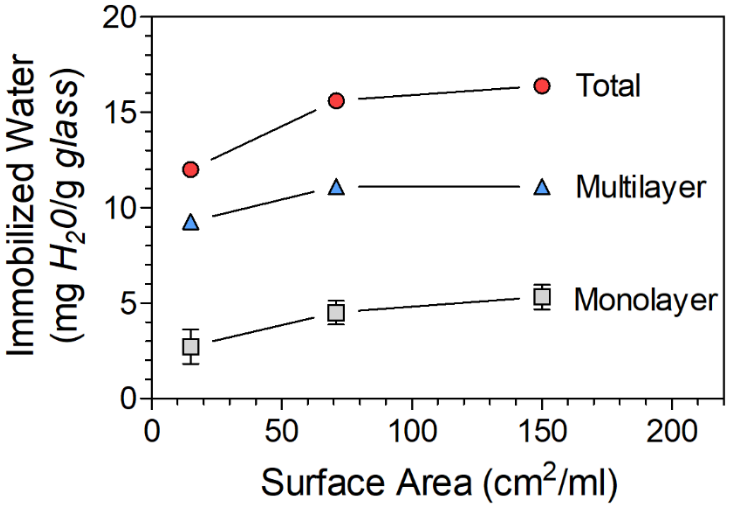

Figure S1 Effect of glass bead surface area on the content of immobilized water in glass bead dispersions. 1% v/v dispersions were prepared in deionized water using glass beads of varying size (4, 7, and 40 µm). Monolayer coverage (gray squares) was calculated from water activity data using the GAB isotherm model (see Methods). Total immobilized water (red circles) was calculated from relative changes in the self-diffusion coefficient of water relative to 0.5% xanthan gum in water. Multilayer immobilized water was estimated by taking the difference between Total and Monolayer water values.

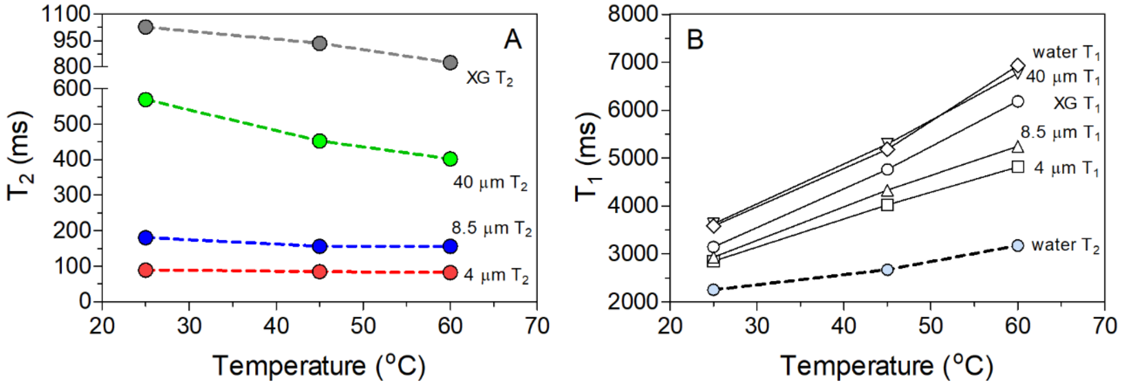

Figure S2 Changes in T_2 and T_1 values at increasing temperature for pure deionized water, 0.5% xantham gum in deionized water, and 1% dispersions of glass microspheres of different sizes in 0.5 wt% xanthan solution. All measurements were conducted at 300 MHz NMR field strength, and temperature was stabilized for 10 min prior to each experiment.

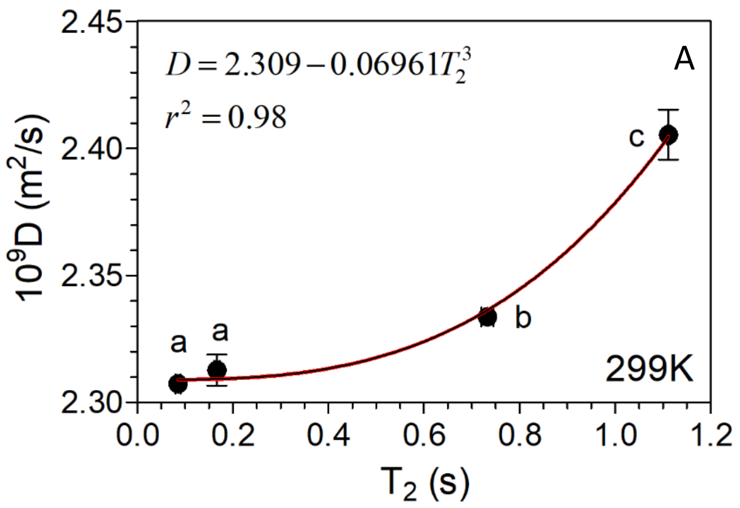

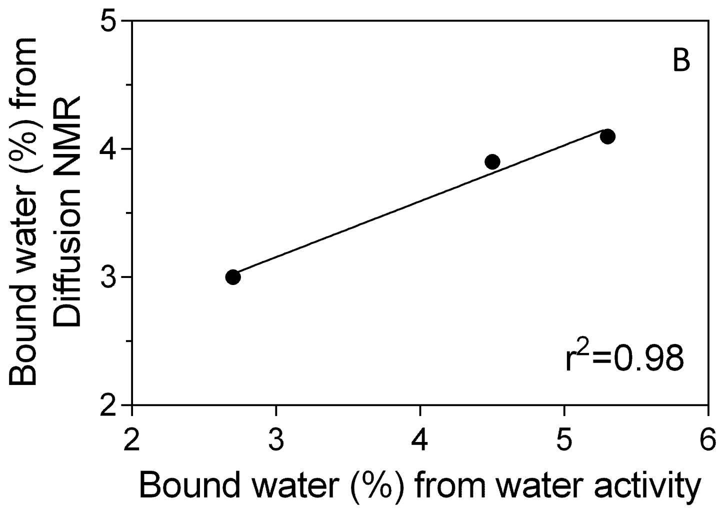

Figure S3 (A) Water diffusion coefficient as a function of T_2 relaxation time for glass microspheres dispersed in a 0.5% xanthan gum solution in deionized water. Samples evaluated were a control (XG solution only), 4 µm, 7 µm, and 40 µm glass beads (1% v/v). All measurements were conducted at 600 MHz NMR field strength, and a sample temperature of 298K. Error bars indicate the SEM. (B) Correlation between the amount of bound water determined by Diffusion NMR and from water activity measurements.

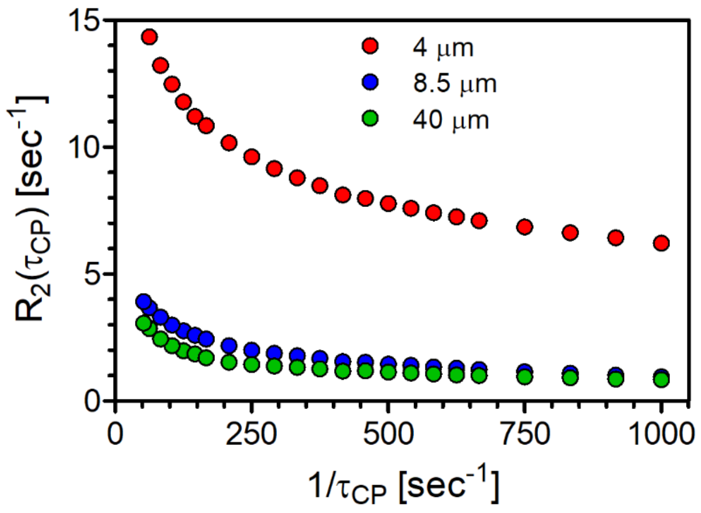

Figure S4 NMR transverse relaxation dispersion profiles of 1% v/v dispersions of 4 µm, 7 µm, and 40 µm glass microspheres (see legend). Dispersions were prepared using a 0.5% xanthan gum solution prepared in deionized water. Plot depicts the transverse relaxation rate ($R_2=1/T_2$) as a function of the interpulse spacing (τ_{CP}) acquired from a series of CPMG pulse sequences at varying τ_{CP} (CPMG-RD experiment; see Methods). All measurements were conducted at 300 MHz NMR field strength, and a sample temperature of 298K.

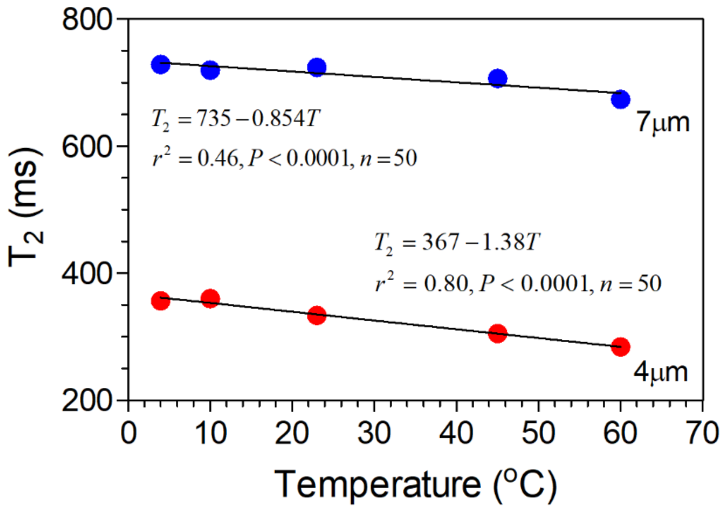

Figure S5 T_2 relaxation times as a function of temperature for 1% v/v dispersions of 4 µm (red) and 7 µm (blue) acid washed glass microspheres prepared in a 0.5% xanthan gum solution. All measurements were conducted at 20 MHz NMR field strength, and a sample temperature of 298K. The experimental data was fit to a simple linear function, which are presented with associated error statistics.

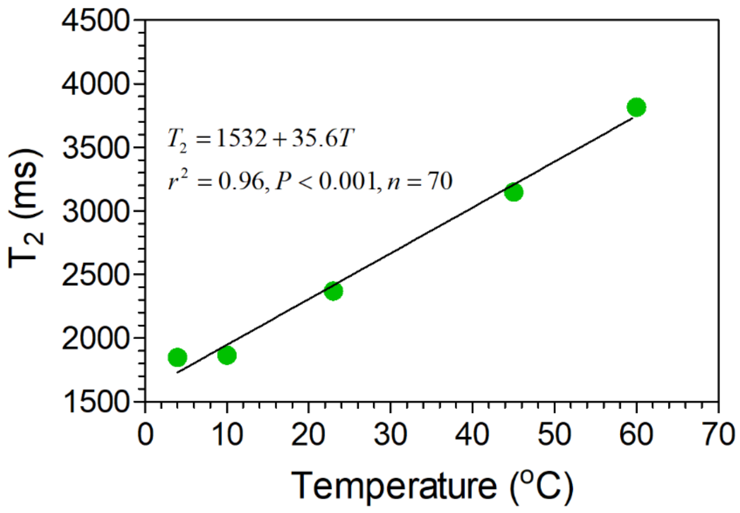

Figure S6 T_2 relaxation times of pure deionized water as a function of temperature. All measurements were conducted at 20 MHz field strength, and a sample temperature of 298K. The experimental data was fit to a simple linear function, which is presented with associated error statistics.

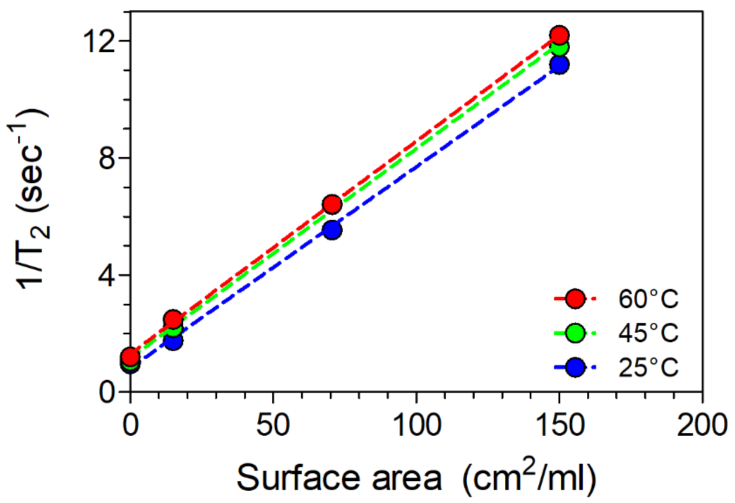

Figure S7 Relaxation rate constants (R_2=1/T_2) for 1% (v/v) suspensions of glass microspheres in 0.5% xanthan gum at different temperatures. Data was normalized to surface area in the suspension.

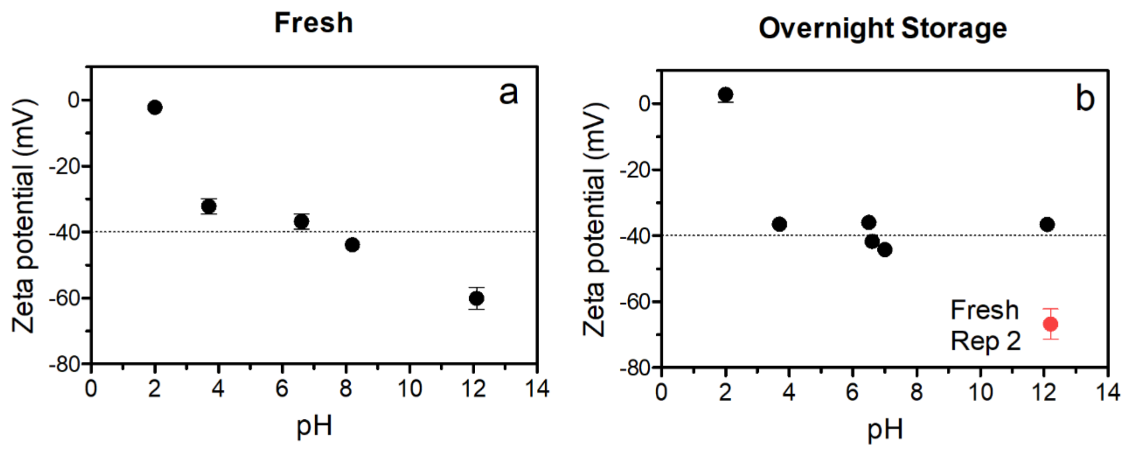

Figure S8 Zeta potential of 4 µm glass microspheres (1% v/v) dispersed in 0.5% xanthan gum, as a function of pH. Dispersions were measured immediately after pH correction (a), and after 24 hours storage (b). Note the red point in (b) was an additional replicate prepared fresh to ensure the initial Zeta potential at pH 12 (Panel A) was not due to experimental error. Error bars indicate the SEM.

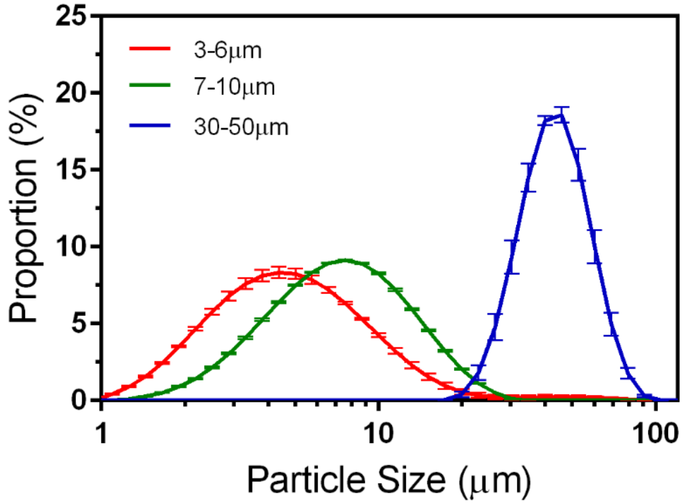

Figure S9 Size distribution of glass microspheres used in this study and their corresponding reported size range. Size distribution was determined using static light scattering through a flow-through cell. Values represent means and standard deviations of n=3 samples.

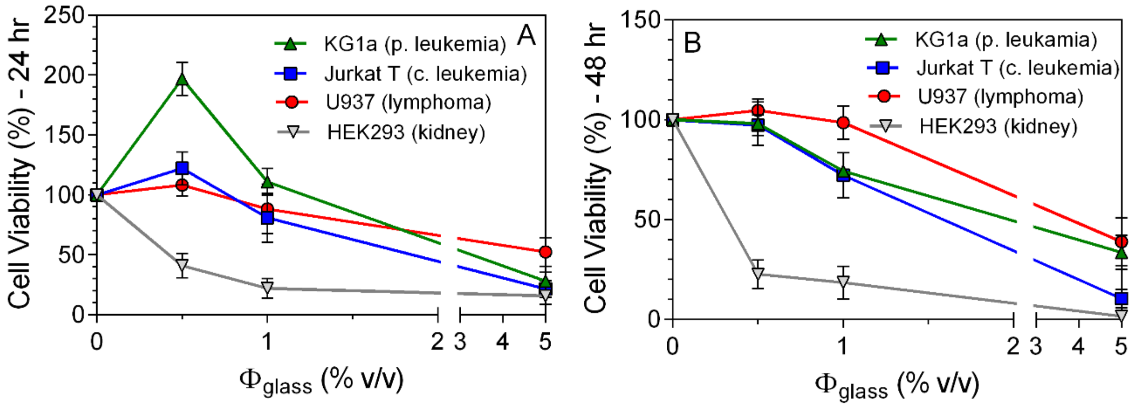

Figure S10 Viability of human lymphoma, leukemia and embryonic kidney cell lines after a 24 hr (A) and 48 hr (B) incubation period in media supplemented with 4 µm glass microspheres (denoted as % v/v glass). Error bars indicate the SEM.

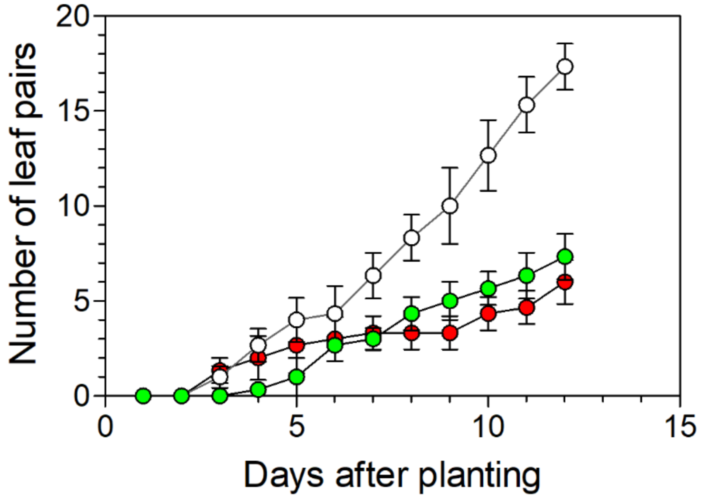

Figure S11 Number of leaf pairs emerging from alfalfa seed sprouts grown in deionized water (white), and water supplemented with 5% v/v 4 µm (green) or 40 µm (red) acid washed glass microspheres. Each sprouting dish contained 40 seeds which were grown over a 12-day period. Error bars indicate the SEM.

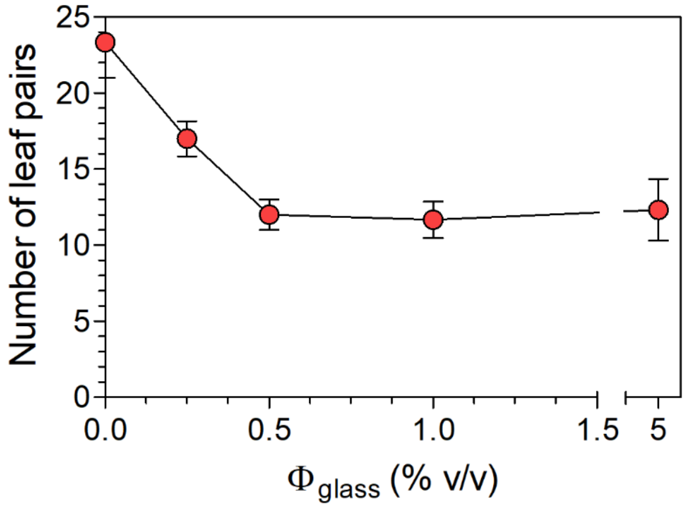

Figure S12 Number of leaf pairs emerging from alfalfa seed sprouts after being grown for 12 days in deionized water and supplemented with an increasing content of acid-washed 4 µm glass microspheres (expressed as % v/v). Each sprouting dish contained 40 seeds. Error bars indicate the SEM.

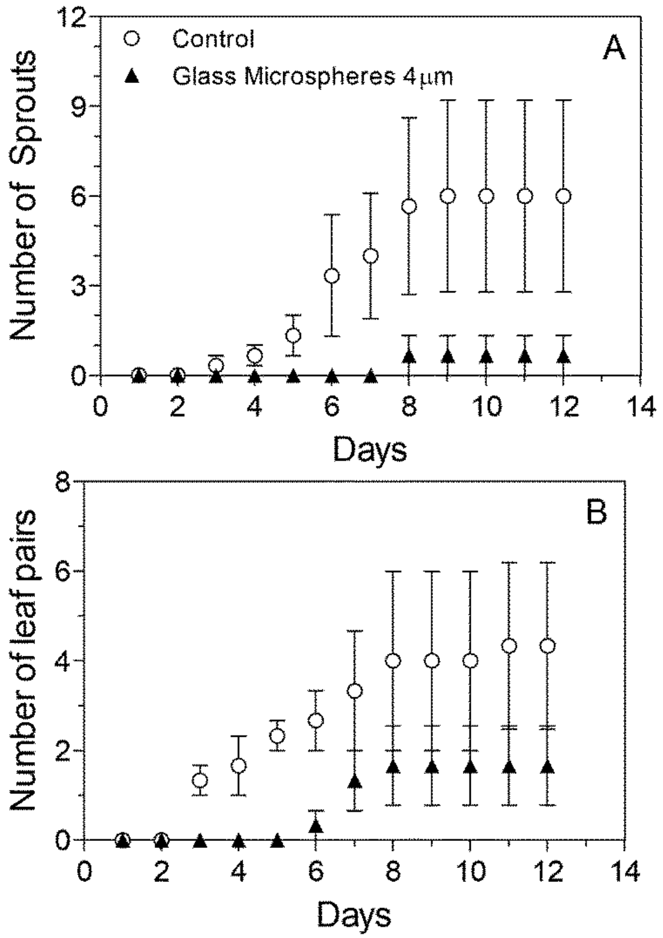

Figure S13 Number of germinated sprouts (A) and leaf pairs (B) emerging from alfalfa seeds grown in a Gamborg's B-5 Basal Broth with Mineral Organics (white circles), and the same medium supplemented with 5% v/v 4 µm glass microspheres (black triangles). Each sprouting dish contained 40 seeds which were grown over a 12 day period. Error bars indicate the SEM.
